# Effect of Inactivated Poliovirus Vaccine Campaigns, Pakistan, 2014–2017

**DOI:** 10.3201/eid2411.180050

**Published:** 2018-11

**Authors:** Nicholas C. Grassly, Mufti Zubair Wadood, Rana M. Safdar, Abdirahman Sheikh Mahamud, Roland W. Sutter

**Affiliations:** Imperial College London, London, United Kingdom (N.C. Grassly);; World Health Organization, Geneva, Switzerland (M.Z. Wadood, R.W. Sutter);; Ministry of National Health Services, Islamabad, Pakistan (R.M. Safdar);; World Health Organization, Islamabad (A.S. Mahamud)

**Keywords:** disease eradication, poliomyelitis, poliovirus vaccine, inactivated, public health, viruses, Pakistan, vaccines, polio, poliovirus

## Abstract

Pakistan began using inactivated poliovirus vaccine alongside oral vaccine in mass campaigns to accelerate eradication of wild-type poliovirus in 2014. Using case-based and environmental surveillance data for January 2014–October 2017, we found that these campaigns reduced wild-type poliovirus detection more than campaigns that used only oral vaccine.

Routine immunization with >1 dose of inactivated poliovirus vaccine (IPV) in all countries using oral poliovirus vaccine (OPV) was recommended by the World Health Organization (WHO) in November 2012, before the global withdrawal of the serotype 2 component from OPV (*1*). IPV has also been used since 2014 in mass campaigns to help interrupt wild poliovirus transmission and stop serotype 2 vaccine-derived poliovirus (VDPV2) outbreaks. The IPV supply was severely constrained during 2016–2017; only 2 manufacturers supply the United Nations Children’s Fund, and their failure to produce the expected bulk product has meant that only about half the awarded quantities were supplied (*2*). As a result of these unplanned reductions in IPV supply, countries have delayed the introduction of IPV to routine immunization or faced stockouts, and mass campaigns with IPV in response to VDPV2 are no longer recommended by WHO (*3*). Nonetheless, where possible, IPV continues to be used in mass campaigns for outbreak response; for example, Pakistan, Afghanistan, Nigeria, and Syria all used IPV in mass campaigns in 2017.

Given that IPV supply constraints are likely to continue until at least the end of 2018, it is crucial that available IPV be optimally allocated between routine immunization and mass campaigns. We recently published estimates of the impact of OPV mass campaigns with and without the inclusion of IPV in Nigeria and Pakistan during January 2014–April 2016 (*4*). These estimates demonstrated a reduction in the incidence of poliomyelitis and detection of poliovirus in the environment after campaigns that used IPV in Nigeria but not in Pakistan, where statistical power was limited. We have now updated these estimates in Pakistan for January 2014–October 2017, thereby including a longer period of surveillance and additional campaigns during a period when wild-type 1 poliovirus has been circulating (Technical Appendix). We find evidence of an impact of campaigns that used IPV alongside OPV (bivalent, trivalent, or monovalent) on the incidence of poliomyelitis caused by wild-type poliovirus (incidence rate ratio [IRR] for 90 days after compared with before the campaign, IRR 0.62, 90% bootstrap CI 0.23–1.14), and a significant impact on the detection of this virus in environmental samples (prevalence ratio [PR] 0.63, 90% CI 0.47–0.81) (Figure; Technical Appendix Table). The effect of campaigns using only bivalent OPV was less than the effect of campaigns that included IPV (IRR for poliomyelitis 0.79 [90% CI 0.64–0.98] and PR for environmental detection 0.92 [90% CI 0.83–1.00] for the 90 days after compared with before the campaign); this difference was statistically significant for detection of poliovirus in the environment (bootstrap p values 0.239 comparing the IRRs and 0.005 comparing the PRs for campaigns with and without IPV). We did not update estimates for Nigeria because only 2 campaigns using IPV occurred during April 2016–October 2017, in areas with very limited VDPV2 detection.

**Figure:**
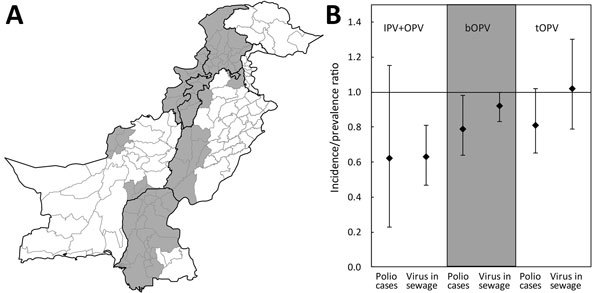
Location and impact of mass campaigns in Pakistan during January 2014–October 2017 that have included inactivated poliovirus vaccine (IPV) alongside oral vaccine. A) Gray shading indicates districts in Pakistan that conducted campaigns with IPV during January 2014–October 2017. B) The incidence rate ratio (IRR) for poliomyelitis and the prevalence ratio (PR) for poliovirus detection in environmental samples (sewage) during 90 days after compared with 90 days before mass vaccination campaigns with different vaccines. The mean estimates (diamonds) are shown with 90% bootstrap CIs (error bars). Detailed methods and results are given in the Technical Appendix. bOPV, bivalent oral poliovirus vaccine; IPV, inactivated poliovirus vaccine; tOPV, trivalent oral poliovirus vaccine.

Several caveats relate to this analysis, reflecting its observational nature, reliance on routinely collected data, and lack of randomization. Campaigns that included IPV may have been implemented with different standards and, potentially, greater coverage, although data supporting this assertion have not been presented. It is often assumed that campaigns including IPV would have lower coverage because IPV must be administered by trained healthcare staff from fixed points rather than in house-to-house campaigns (*5*). Furthermore, these findings may not apply to more recent serotype-2 vaccine-derived poliovirus outbreaks, which have occurred in countries without recent use of a serotype-2–containing oral vaccine, thereby limiting boosting of mucosal immunity by IPV to older cohorts.

In conclusion, these updated estimates from Pakistan provide support for including IPV in mass campaigns with OPV to reduce poliovirus transmission, in agreement with results from Nigeria. Intradermal administration of a 1/5 fractional dose may allow dose sparing during these campaigns while maintaining comparable immunogenicity (*6*). These findings are informing discussions about the role of IPV in stopping the last remaining chains of wild-type 1 poliovirus transmission, responding to VDPV2 outbreaks, and protecting children who have not received vaccine containing serotype 2.

Technical Appendix Discussion of data and statistical analysis methods for the study of poliovirus vaccine campaigns, Pakistan, 2014–2017.
