# Supplementary material for: Effect of Inactivated Poliovirus Vaccine Campaigns, Pakistan, 2014–2017
Source: Emerg Infect Dis. 2018 Nov;24(11):2113–5. doi: 10.3201/eid2411.180050 (PMC6200014; doi:10.3201/eid2411.180050)
Supplement: Technical Appendix — Discussion of data and statistical analysis methods for the study of poliovirus vaccine campaigns, Pakistan, 2014–2017. [file 18-0050-Techapp-s1.pdf]

Article DOI: https://doi.org/10.3201/eid2411.180050

Effect of Inactivated Poliovirus Vaccine Campaigns, Pakistan, 2014–2017

Technical Appendix

Data

Pakistan implements acute flaccid paralysis and environmental surveillance for polioviruses (*1*). We extracted these surveillance data together with the vaccination campaign calendar for the period January 2014–October 2017 from the Polio Information System (PolIS) on December 16, 2017. Campaigns with inactivated poliovirus vaccine (IPV) and oral poliovirus vaccine (OPV) recorded as occurring within 14 days were considered the same campaign in this analysis (i.e., IPV+OPV). Administrative boundaries for the map in the Figure in the print article were provided by the World Health Organization. The publication of this map does not imply the expression of any opinion whatsoever on the part of WHO concerning the legal status of any territory, city, or area or of its authorities, or concerning the delimitation of its frontiers or boundaries. Estimates of the number of children 0–14 years of age living in each district were obtained as described previously (*2*).

Statistical Analysis

We calculated the incidence of poliomyelitis associated with serotype-1 wild poliovirus and the prevalence of this virus in environmental samples in the 90 days before and after mass campaigns using different vaccine types in each district of Pakistan. We used mixed-effects Poisson or binomial regression to estimate the incidence rate ratio (IRR) or prevalence ratio (PR) for poliomyelitis incidence or environmental detection of poliovirus after a campaign compared with before the campaign. Full details of the methods are given by Shirreff et al. (*2*). When 2 or more campaigns took place in a district within 180 days such that the observation periods overlapped, we censored the data to avoid the inclusion of nonindependent (repeated) data in the

analysis. We randomly selected the order in which districts and campaigns were included in the analysis, prioritizing campaigns that included IPV. This meant that some campaigns were associated with <180 days observation or were not included in the database. Here we report a range for the number of campaigns, incidence of poliomyelitis, and prevalence in environmental samples based on 1,000 repetitions of this process. The IRR and PR were estimated along with their 90% confidence intervals based on the mean and the 5th and 95th percentiles for their values for 1,000 bootstrap replicates of the data, sampling with replacement (i.e., without censoring) to describe the sampling distribution of these statistics following standard procedures (*3*).

References

1. Elhamidi Y, Mahamud A, Safdar M, Al Tamimi W, Jorba J, Mbaeyi C, et al. Progress toward poliomyelitis eradication—Pakistan, January 2016–September 2017. MMWR Morb Mortal Wkly Rep. 2017;66:1276–80. PubMed http://dx.doi.org/10.15585/mmwr.mm6646a4

2. Shirreff G, Wadood MZ, Vaz RG, Sutter RW, Grassly NC. Estimated effect of inactivated poliovirus vaccine campaigns, Nigeria and Pakistan, January 2014–April 2016. Emerg Infect Dis. 2017;23:258–63. PubMed http://dx.doi.org/10.3201/eid2302.161210

3. Efron B, Tibshirani R. Bootstrap methods for standard errors, confidence intervals, and other measures of statistical accuracy. Stat Sci. 1986;1:54–75. http://dx.doi.org/10.1214/ss/1177013815

Technical Appendix Table. Incidence of poliomyelitis associated with wild-type 1 poliovirus and prevalence of this virus in environmental samples in Pakistan in the 90-day period before and after campaigns with different vaccine types.*

Vaccine	State	District campaigns included in analysis (n)†	Range of incidence of poliomyelitis (n cases/100,000 child-years)‡ Before	After	IRR (90% bootstrap CI)	Prevalence in environmental samples, range (%) Before	After	PR (90% bootstrap CI)
IPV+ OPV	All	166	(17–28)/ (162–165)	(7–17)/ (158–160)	0.62 (0.23–1.15)	25 (24–26)	15 (14–16)	0.63 (0.47–0.81)
	Balochistan	14	(10–15)/ (9.53–10.8)	(2,4)/ (9.53–10.8)		45 (42–48)	25 (22–28)	
	FATA	17	(1,2)/ (6.25,6.25)	(0,6)/ (6.25,6.25)		NA	NA	
	Gilgit Baltistan	0	0/0	0/0		NA	NA	
	Islamabad	2	0/ (1.65–1.65)	0/ (0.33–0.33)		0 (0–0)	NA	
	Khyber Pakhtunkhwa	57	(5–10)/ (59.6–59.6)	(5,7)/ (59.6–59.6)		25 (22–25)	8 (8–8)	
	Punjab	9	0/ (26.1–26.1)	0/ (23.1–23.1)		14 (14–14)	21 (21–21)	
	Sindh	67	(1–2)/ (58.8–60.3)	0/ (58.8–60.3)		16 (15–16)	11 (11–12)	
bOPV	All	1330–1392	(102–192)/ (997–1081)	(77–165)/ (962–1042)	0.79 (0.64–0.98)	20 (17–24)	20 (15–23)	0.92 (0.83–1.00)
	Balochistan	241–265	(5–14)/ (39.3–49.2)	(4–13)/ (38.3–47.4)		31 (21–41)	27 (16–38)	
	FATA	107–124	(31–103)/ (21.6–28.5)	(35–91)/ (20.7–26.8)		NA	NA	
	Gilgit Baltistan	53–63	(0–1)/ (4.76–5.98)	(0–1)/ (4.87–6.06)		NA	NA	
	Islamabad	12–18	0/ (7.04–11.8)	0/ (7.25–12.9)		22 (6–38)	19 (5–33)	
	Khyber Pakhtunkhwa	172–195	(20–53)/ (110–134)	(12–43)/ (99.8–122)		15 (9–23)	19 (8–30)	
	Punjab	296–324	(2,6)/ (561–630)	(0–3)/ (554–629)		11 (8–15)	9 (5–13)	
	Sindh	324–349	(19–33)/ (207–237)	(13–28)/ (185–212)		31 (24–38)	33 (25–41)	
tOPV	All	81–105	(3–62)/ (45.5–90.1)	(0–30)/ (39.5–72.6)	0.81 (0.65–1.02)	7 (6–20)	20 (8–50)	1.02 (0.79–1.30)
	Balochistan	22–27	0/ (1.81–4.99)	(0–2)/ (1.81–3.96)		NA	NA	
	FATA	0–8	(0–55)/ (0–1.23)	(0–23)/ (0–1.37)		NA	NA	
	Gilgit Baltistan	7–7	0/ (0.15–0.86)	0/ (0.25–0.83)		NA	NA	
	Islamabad	0–2	0/ (0–1.65)	0/ (0–1.18)		0 (0–0)	0 (0–0)	
	Khyber Pakhtunkhwa	6–16	(0–5)/ (3.70–15.84)	(0–5)/ (2.03–9.01)		NA	NA	
	Punjab	21–31	(0–2)/ (22.71–62.13)	0/ (22.3–54.2)		0 (0–0)	0 (0–0)	
	Sindh	4–16	0/ (2.03–10.46)	(0–1)/ (1.48–11.2)		33 (17–33)	100 (67–100)	

*IRR = incidence rate ratio; NA, not available; PR = prevalence ratio.
†Counts campaigns in each district separately (e.g., a campaign covering 5 districts would be counted as 5 district campaigns). When calculating the number of campaigns, incidence, and prevalence, we included only those providing independent data after-censoring observations with overlapping tim periods (see Methods in the print article and this Technical Appendix). The order of censoring can affect this number, so we randomly added campaigns to the analysis database, prioritizing those that included IPV. We show the range from 1,000 replicates of this random selection procedure. The number of campaigns with IPV did not vary because these were prioritized in the censoring process, so only a single number is shown.
‡Child-years are based on estimates of children aged 0–14 y, corresponding to the age range for AFP surveillance. The range in the number of cases and child-years included in the analysis for each random sample of the data are shown in the numerator and denominator, respectively.
